# Comparing the effects of HIV self-testing to standard HIV testing for key populations: a systematic review and meta-analysis

**DOI:** 10.1186/s12916-020-01835-z

**Published:** 2020-12-03

**Authors:** T. Charles Witzel, Ingrid Eshun-Wilson, Muhammad S. Jamil, Nerissa Tilouche, Carmen Figueroa, Cheryl C. Johnson, David Reid, Rachel Baggaley, Nandi Siegfried, Fiona M. Burns, Alison J. Rodger, Peter Weatherburn

**Affiliations:** 1 Department of Public Health, Environments and Society, London School of Hygiene & Tropical Medicine, 15-17 Tavistock Place, London, WC1H 9SH UK; 2 Washington University School of Medicine, St Louis, USA; 3 Global HIV, Hepatitis and STI Programme, World Health Organization, Geneva, Switzerland; 4 Independent Clinical Epidemiologist, Cape Town, Republic of South Africa; 5 Institute for Global Health, University College London, London, UK

**Keywords:** HIV self-testing, Men who have sex with men, Trans people, Female sex workers, Meta-analysis, HIV prevention, HIV testing

## Abstract

**Background:**

We update a previous systematic review to inform new World Health Organization HIV self-testing (HIVST) recommendations. We compared the effects of HIVST to standard HIV testing services to understand which service delivery models are effective for key populations.

**Methods:**

We did a systematic review of randomised controlled trials (RCTs) which compared HIVST to standard HIV testing in key populations, published from 1 January 2006 to 4 June 2019 in PubMed, Embase, Global Index Medicus, Social Policy and Practice, PsycINFO, Health Management Information Consortium, EBSCO CINAHL Plus, Cochrane Library and Web of Science. We extracted study characteristic and outcome data and conducted risk of bias assessments using the Cochrane ROB tool version 1. Random effects meta-analyses were conducted, and pooled effect estimates were assessed along with other evidence characteristics to determine the overall strength of the evidence using GRADE methodology.

**Results:**

After screening 5909 titles and abstracts, we identified 10 RCTs which reported on testing outcomes. These included 9679 participants, of whom 5486 were men who have sex with men (MSM), 72 were trans people and 4121 were female sex workers. Service delivery models included facility-based, online/mail and peer distribution. Support components were highly diverse and ranged from helplines to training and supervision. HIVST increased testing uptake by 1.45 times (RR=1.45 95% CI 1.20, 1.75). For MSM and small numbers of trans people, HIVST increased the mean number of HIV tests by 2.56 over follow-up (mean difference = 2.56; 95% CI 1.24, 3.88). There was no difference between HIVST and SoC in regard to positivity among tested overall (RR = 0.91; 95% CI 0.73, 1.15); in sensitivity analysis of positivity among randomised HIVST identified significantly more HIV infections among MSM and trans people (RR = 2.21; 95% CI 1.20, 4.08) and in online/mail distribution systems (RR = 2.21; 95% CI 1.14, 4.32). Yield of positive results in FSW was not significantly different between HIVST and SoC. HIVST reduced linkage to care by 17% compared to SoC overall (RR = 0.83; 95% CI 0.74, 0.92). Impacts on STI testing were mixed; two RCTs showed no decreases in STI testing while one showed significantly lower STI testing in the intervention arm. There were no negative impacts on condom use (RR = 0.95; 95% CI 0.83, 1.08), and social harm was very rare.

**Conclusions:**

HIVST is safe and increases testing uptake and frequency as well as yield of positive results for MSM and trans people without negative effects on linkage to HIV care, STI testing, condom use or social harm. Testing uptake was increased for FSW, yield of positive results were not and linkage to HIV care was worse. Strategies to improve linkage to care outcomes for both groups are crucial for effective roll-out.

**Supplementary information:**

The online version contains supplementary material available at 10.1186/s12916-020-01835-z.

## Background

There has been significant progress and scale-up of HIV testing services in the past decade. In 2018, it was estimated that globally 79% of people with HIV were aware of their status, the majority of whom were on treatment and achieving viral suppression [1]. Despite this progress, more than 5 million people with HIV remain undiagnosed [1].

Undiagnosed and untreated HIV infections contribute to the majority of new infections. Studies in the USA and UK suggest that between 60 and 80% of new infections were transmitted by people who did not know their status, or who were not yet on treatment [2, 3].

Key populations are disproportionately impacted by HIV despite making up small proportions of the overall population. Approximately 54% of all new infections were among key populations (men who have sex with men (MSM), people who inject drugs (PWID), people in prisons and other closed settings, sex workers and trans people) and their partners in 2018. Increasing HIV testing, prevention and treatment coverage, along with viral suppression, among these groups is a global health priority.

HIV self-testing (HIVST), whereby a person collects their own sample, performs a rapid test and then interprets their own result, has been highlighted as an important approach for reaching key populations [4–7]. Because of its convenient and private nature, HIVST interventions have the potential to increase uptake and frequency of testing among those less likely to test through other mechanisms by overcoming structural and individual barriers including direct and opportunity costs, as well as fear of stigma and discrimination [6, 8–12]. By reaching those less likely to test, HIVST may lead to the detection of greater numbers of previously undiagnosed infections when compared to standard approaches.

While there has been longstanding interest in HIVST, implementation is relatively new. Following the completion of five randomised controlled trials (RCTs) in addition to large-scale country evaluations, in 2016, the World Health Organization (WHO) recommended HIVST as an additional testing approach [13]. This guidance was based on evidence showing that HIVST was a safe and effective way to increase testing uptake and frequency.

As of June 2019, 77 countries had supportive policies, but HIVST had only been implemented in 38 [14]. To facilitate scale-up of HIVST, it is essential to understand which service delivery approaches are the most safe, acceptable and effective for reaching different key populations.

This review aims to compare the effects of HIVST to standard HIV testing and to understand which HIVST service delivery models are effective for key populations. It was conducted to update the WHO guidelines and recommendations on HIVST. In doing so, we updated a previous systematic review and meta-analysis, in order to capture significant development in the evidence base since that prior review [15]. This is one among a series of reviews examining outcomes for general populations, key populations and intervention values and preferences of HIVST users as well as a network meta-analysis.

## Methods

This systematic review was conducted in line with the PRISMA guidelines for systematic reviews and meta-analyses [16].

### Searches, screening and data extraction

Our review followed a PICO question (see Table 1) which was determined as part of the 2019 WHO HIVST guidelines development process [17, 18]. We limited eligibility to RCTs reporting on one of more of our outcomes among at least one key population and which sought to compare HIVST against any other HIV testing intervention, referred to as standard of care (SoC) [19]. We had no limits on language but only included literature published in academic journals and conferences. The full review protocol, including search details, is available in Additional file 1.

**Table 1:** Review PICO

**Population**	Key populations receiving HIV testing services
**Intervention**	Interventions which provide HIVST
**Comparison**	HIV testing interventions which do not use HIVST
**Outcomes**	HIV testing uptake, HIV testing frequency, STI testing frequency, condom use, HIV positivity, linkage to care, adverse events

The search strategy was previously validated for systematic mapping of HIVST literature [18]. Databases searched include PubMed, Embase, Global Index Medicus, Social Policy and Practice, PsycINFO, Health Management Information Consortium, EBSCO CINAHL Plus, Cochrane Library and Web of Science [18]. These were first searched from 1 January 2006 to 1 January 2016 and then updated monthly until 4 June 2019. Conference abstract searches included African Society for Laboratory Medicine Conference (ASLM), Conference on Retroviruses and Opportunistic Infections (CROI), International AIDS Conference and International AIDS Society Conference (IAS). For CROI, only the most recent conferences (2014–2019) were searched as past conferences are inaccessible. We also searched AIDS Impact 2019.

Titles and abstracts were reviewed in duplicate by the first author and other members of the study team (CF, DR, NT, MSJ). These were then screened by two researchers (TCW and MSJ) for inclusion. Authors of one study (MacGowan et al. [20]) were aware of this meta-analysis following requests for additional data from an earlier conference presentation. They alerted us to the published manuscript with updated analyses, which we included for completeness.

Outcome data were extracted in duplicate by two researchers (TCW and one of NT, MSJ, IE-W) using standardised extraction forms and entered into a relational database tool (airtable.com). Disagreements were resolved by consensus. Where data were not available, authors were contacted for additional information.

The outcomes included (1) uptake of HIV testing, (2) frequency of HIV testing (mean number of tests over a period of time), (3) HIV positivity (positive results/all tested and positive results/all randomised), (4) linkage to treatment or care, (5) uptake and frequency of sexually transmitted infections (STI) testing, (6) condom use and (7) social harm or adverse events. Linkage was a binary variable with any linkage to care or ART initiation as reported by authors. For positivity, we conducted an analysis of test positivity (proportion of positive results among those tested) and a sensitivity analysis using the number of participants randomised as denominator (intention-to-treat approach). All studies including trans people grouped this population with MSM; we therefore report these two groups as one.

### Quality assessment

The Cochrane risk of bias tool was used to evaluate studies for methodological quality [21]. This included evaluation of risk of bias pertaining to random sequence generation, allocation concealment, blinding of participants and personnel, attrition bias and reporting bias [21]. Publication bias was assessed with forest plots when there were sufficient studies to do so (*n* > 10).

We followed GRADE methodology to assess the certainty of the evidence for each outcome across GRADE domains: methodological quality, imprecision, indirectness, inconsistency and publication bias [17, 19]. Heterogeneity was defined as either low (*i*^2^ < 25%), medium (*i*^2^ = 25–75%) or high (*i*^2^ > 75%).

### Data analysis

Where more than two studies reported the same or a comparable outcome, a meta-analysis was conducted. All meta-analyses were conducted using random effects models (inverse-variance method) in RevMan 5.4. For dichotomous outcomes, risk ratios (RRs) and 95% confidence intervals (CIs) were calculated and pooled. For continuous outcomes, mean differences with 95% CIs were calculated and pooled. Statistical heterogeneity was evaluated using the DerSimonian-Laird estimator for Tau^2^ and the associated *I*^2^ statistic. For each outcome within the meta-analysis, we generated forest plots overall and by each stratification. Where possible, we performed sub-group analyses on key population group (MSM and trans people; FSW) and by service delivery model (facility-based; online/mail; peer distribution). When outcomes were measured and reported at multiple timepoints, we used the longest timepoint where possible. For two studies, we used the earliest timepoints for both uptake and positivity because uptake data were not cumulatively reported from multiple timepoints whereas positivity data were, prohibiting comparison at later timepoints.

For cluster RCTs, we included cluster-adjusted RRs and CIs as reported by the authors where possible. 

For RCTs with multiple intervention arms (1) data from different intervention arms were combined where reviewers assessed the interventions as unlikely to influence the outcome, and (2) where reviewers assessed the interventions as likely to influence the outcome, the intervention arms were not combined for delivery model sub-group analyses. Where one of the intervention arms was an enhanced or optimised version of control arm, we did not include it in meta-analysis.

We did not perform sub-group analyses by support tools (e.g. online counselling; enhanced instructions) used in the RCTs as they were highly heterogenous with very small numbers in each category.

This systematic review was not registered as it was part of an internal endeavour commissioned by the WHO to update their normative HIVST guidance.

## Results

**Fig. 1:**
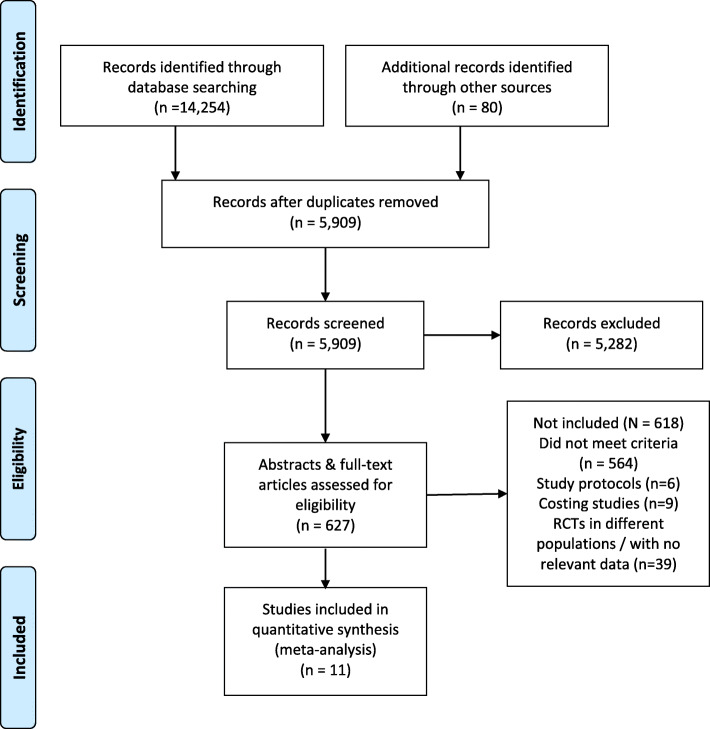
Flowchart of the selection of studies

We identified 14,254 records from databases, 77 conference abstracts and 3 from other sources. After duplicates were removed, 5909 titles and abstracts were screened for eligibility. We screened 627 full-text articles for eligibility. Eleven studies reporting results from 10 RCTs met inclusion criteria. See Fig. 1 for full PRISMA diagram and Additional file 2 for risk of bias assessments.

The ten RCTs enrolled 9679 participants of whom 5486 were MSM, 72 were trans people (mostly trans women) and 4121 were FSW. No trials among people who inject drugs or people in prisons or closed settings were identified. Table 2 provides summaries of included studies and details of support tools used in the RCTs. All studies included only participants with negative or unknown HIV status.

**Table 2:** Characteristics of included RCTs

Study	Country	Total randomised	Study population	Intervention(s)/support tools	Standard of care	HIVST distribution method
Chanda et al. [22]	Zambia	965	FSW	• Peer educator provided risk reduction counselling; condom distribution; HIV testing information • Group HIVST demonstration • Peer educator delivered HIVST kit (arm 1) • Peer educator delivered coupons for HIVST collection at facilities (arm 2)	• Peer educator provided risk reduction counselling; condom distribution; HIV testing information	• Facility-based distribution (arm 1) • Secondary distribution—peers (arm 2)
Jamil et al. [23]	Australia	362	MSM	• 4 HIVST kits at enrolment, option to request additional kits (max 12 per year) • 24/7 telephone hotline	• Standard HIV testing services	• Facility-based distribution • Distribution by mail (optional)
Katz et al. [24]	USA	230	MSM (n=226), trans people (n=4; 1 trans woman, 3 gender-queer/neutral)	• 1 HIVST kit at enrolment, option to request additional kits (max 1 per month) • In-person demonstration; information about HIV testing and reminders; 24/7 telephone hotline	• HIV testing advice • Offer of testing reminders (email, phone or letter) at desired frequency	• Facility-based distribution • Distribution by mail (optional)
Kelvin et al. [25]	Kenya	2196	FSW	• Choice of supervised self-administered HIVST at facility (overseen by health worker) or free HIVST kit for home use	• Provider administered testing • Testing reminder via SMS	• HIVST at facilities
MacGowan et al. [20]	USA	2665	MSM	• 4 HIVST kits at enrolment (mail), option to request additional kits 3 monthly • HIV testing information; 24/7 telephone hotline	• Standard HIV testing services	• Online/mail distribution
Merchant et al. [26]	USA	425	MSM (18–24 years)	• Internet gift card for online order of HIVST kit	• HIV testing advice • Web link to testing service locator	• Online/mail distribution
Ortblad et al. [27, 28]	Uganda	960	FSW	• Peer educator provided risk reduction counselling; condom distribution; HIV testing information • Group HIVST demonstration • Peer educator delivered HIVST kit (arm 1) • Peer educator delivered coupons for HIVST collection at facilities (arm 2)	• Peer educator provided risk reduction counselling; condom distribution; HIV testing information	• Facility-based distribution (arm 1) • Secondary distribution—peers (arm 2)
Tang et al. [29]	China	1381	MSM (n=1313), trans women (n=68)	• Access to HIVST kits promoted via social media along with a promotional campaign on HIV testing	• Routine health promotion efforts	• Online/mail distribution
Wang et al. [30]	Hong Kong SAR	430	MSM	• 1 HIVST kit at enrolment (mail) • 3 min online video promoting HIV testing; 4 min online video promoting HIVST; 15 min motivational interview conducted over the phone by trained staff to promote HIVST • Real-time instructions and pre/post-test counselling provided online • Accompaniment to clinic appointment for confirmatory testing	• 3 min online video promoting HIV testing	• Online/mail distribution
Wray et al. [31]	USA	65	MSM	• HIVST kits mailed at 3 monthly intervals (arm 1) • HIVST kits fitted with Bluetooth device mailed at 3 monthly intervals (arm 2) • Follow-up by counsellor when kit opened; risk reduction counselling and referral to prevention services (arm 2)	• 3-monthly letters with HIV testing information	• Online/mail distribution

Most of the seven studies including MSM were conducted in high-income settings (four in the USA [20, 24, 26, 31], one in Australia [23], one in Hong Kong [30]), except for one RCT in China (a medium high-income setting) [29]. All studies including FSW were conducted in low or lower middle-income settings, all in sub-Saharan Africa (one each in Kenya [25], Uganda [27, 28] and Zambia [22]). All studies used oral fluid-based HIVST kits, except one which provided oral fluid and also fingerprick/blood-based HIVST [20]. All studies provided kits free of charge. Jamil et al. [23], MacGowan et al. [20] and Katz et al. [24] enabled individuals to take more than one kit per person at a time and to collect additional HIVST kits during the study period. Ortblad et al. and Chanda et al. provided a second HIVST in intervention arms at 3 months [22, 27].

The 10 RCTs included 13 HIVST interventions. All compared HIVST against SoC. A range of intervention designs were identified. Most interventions delivered HIVSTs through facilities (*n* = 5) [22–25, 27] or online/mail distribution systems (*n* = 6) [20, 26, 29–31], with a minority (*n* = 2) delivering kits through peers [22, 27]. Both trials distributing through peers targeted FSW only [22, 27].

Support tools were highly diverse. Two interventions delivered to MSM included real-time pre- and post-test counselling, one through a prearranged video appointment system [30] and one through a Bluetooth beacon which activated when the kit was opened prompting a counsellor to contact the user [31]. Three MSM studies offered telephone hotlines [20, 23, 24]. The provision of risk reduction information was included in three studies [22, 27, 31].

Additional file 3 presents a summary of findings including GRADE assessments.

### Uptake of HIV testing

All ten RCTs reported on uptake of HIV testing across 12 HIVST interventions^1^ [20, 22–27, 29–31]. A meta-analysis showed that HIVST increased the uptake of HIV testing by 1.45 times compared to SoC (relative risk (RR) = 1.45; 95% CI 1.20, 1.75; *I*^2^ = 97%; moderate-quality evidence). Eight of the 10 RCTs showed a statistically significant increase in uptake of HIV testing. Publication bias was assessed for this outcome, funnel plots showed benefit from all studies and we assessed this was true intervention impact (see Additional file 4).

Seven of the ten RCTs included data for MSM; two of these also included trans people [20, 23, 24, 26, 29–31]. A sub-group analysis of these showed that HIVST increased the uptake of HIV testing by 1.48 times compared to SoC (RR = 1.48; 95% CI 1.21, 1.81; *I*^2^ = 95%; low-quality evidence).

Three RCTs were conducted among FSW [22, 25, 27]. Our sub-group analysis indicated HIVST increased uptake of testing (RR = 1.36; 95% CI 1.04, 1.78; *I*^2^ = 95%; moderate-quality evidence) (see Additional file 5).

**Fig. 2:**
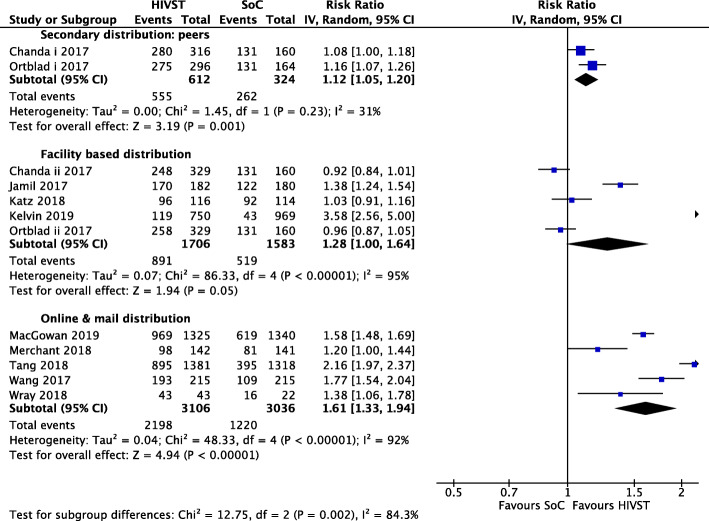
Meta-analysis of studies evaluating HIV testing uptake with delivery mechanism stratification

A sub-group analysis of five interventions delivering HIVST to MSM, trans people and FSW through facilities showed HIVST increased uptake by 1.28 times (RR = 1.28; 95% CI 1.00, 1.64; *I*^2^ = 95%; moderate-quality evidence) [22–25, 27]. Secondary distribution through peers among FSW showed HIVST increased uptake by 1.12 times (RR = 1.12; 95% CI 1.05, 1.20; *I*^2^ = 31%; moderate-quality evidence) [22, 27]. Sub-group analysis of online/mail distribution in MSM and trans people found that HIVST delivered this way increased uptake by 1.61 times (RR = 1.61; 95% CI 1.33, 1.94; *I*^2^ = 92%; moderate-quality evidence) [20, 26, 29–31]. Figure 2 provides delivery method stratification meta-analysis results.

### Frequency of HIV testing

**Fig. 3:**
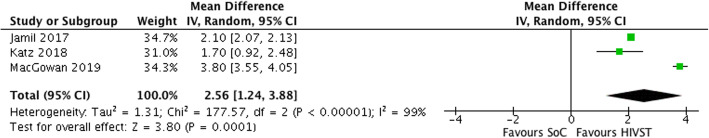
Meta-analysis of studies evaluating HIV testing frequency

A meta-analysis of three RCTs found that HIVST increased mean number of HIV tests by 2.56 during follow-up (mean difference = 2.56; 95% CI 1.24, 3.88; *I*^2^ = 99%; moderate-quality evidence) (Fig. 3); all RCTs showed benefit [20, 23, 24]. Two studies delivered HIVST through facility distribution (with additional, optional mail distribution) and had smaller effect sizes at 2.1 and 1.7, respectively [23, 24]. One delivered HIVST through mail and demonstrated the largest difference at 3.80 [20]. All studies were conducted with MSM with small numbers of trans people also included in one.

### HIV positivity

**Fig. 4:**
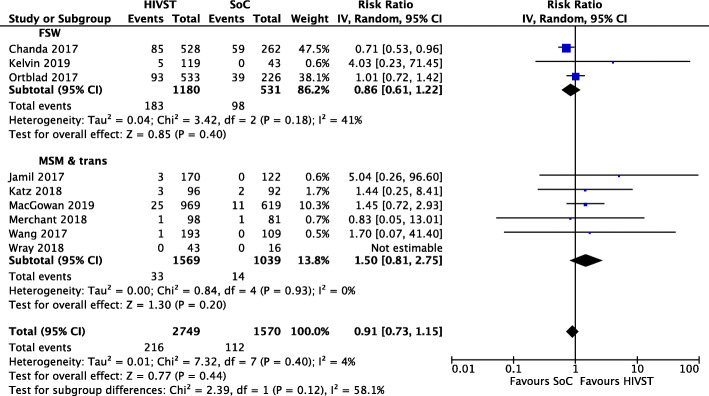
Meta-analysis of studies evaluating HIV positivity among tested with population stratification

Nine of the 10 RCTs reported on positive results among tested. A meta-analysis indicated HIVST had no effect on HIV positivity among those tested (IRR = 0.91; 95% CI 0.73, 1.15; *I*^2^ = 4%; low-quality evidence) [20, 22–27, 30, 31]. No sub-group analyses among population groups showed significant differences (Fig. 4).

A sub-group analysis of delivery mechanisms showed no significant differences in HIV positivity among those tested in online/mail distribution (RR = 1.41; 95% CI 0.73, 2.75; *I*^2^ = 0%; moderate-quality evidence), in facility-based distribution (RR = 0.99, 95% CI 0.59, 1.66; *I*^2^ = 38%; moderate-quality evidence) or in secondary HIVST distribution through peers (RR = 0.78; 95% CI 0.57, 1.06; *I*^2^  =0; low-quality evidence) (forest plot available in Additional file 5).

**Fig. 5:**
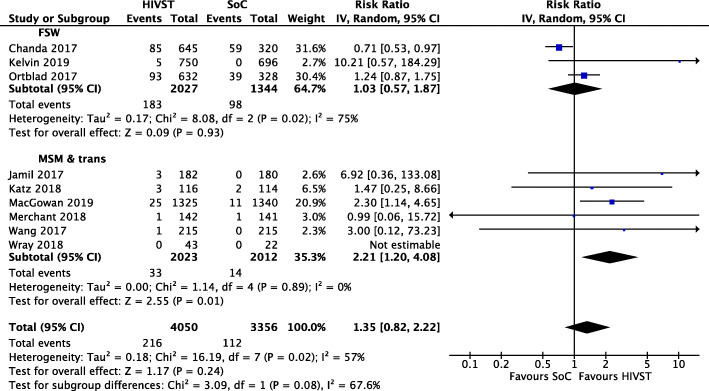
Meta-analysis of studies evaluating HIV positivity among randomised with population stratification

Sensitivity analysis of positivity by *total number randomised* provided different results. Overall differences between HIVST and SoC were not significant. However, in a sub-group analysis of seven studies conducted among MSM (one including trans people) [20, 23, 24, 26, 30, 31], HIVST arms yielded more than double the rate of positivity compared to SoC arms (RR = 2.21; 95% CI 1.20, 4.08; *I*^2^ = 0%; moderate-quality evidence). Significant differences were not seen for FSW (Fig. 5) [22, 25, 27].

In sub-group analyses, online/mail distributed HIVST found increased the rate of positivity among all randomised by 2.21 times compared to SoC (RR = 2.21; 95% CI 1.14, 4.32; *I*^2^ = 0%; moderate-quality evidence). No significant differences were observed in peer or facility-based delivery of HIVST compared to SoC (see Additional file 5 for forest plot).

### Linkage to care

**Fig. 6:**
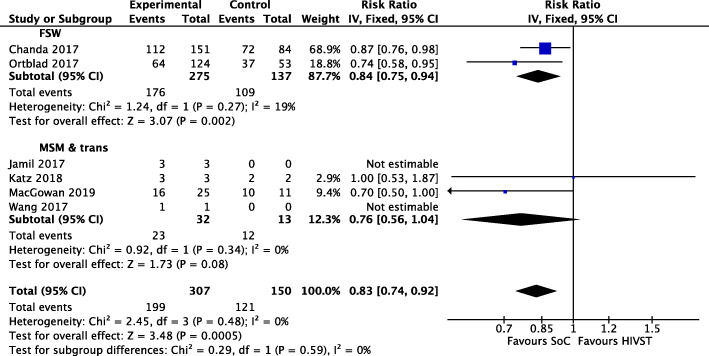
Meta-analysis of studies evaluating linkage to care with population stratification

Six RCTs measured linkage to HIV care or antiretroviral therapy among key populations diagnosed with HIV [20, 22–24, 27, 30]. A meta-analysis of moderate-quality evidence indicated that HIVST reduced linkage to care by 17% (RR = 0.83; 95% CI 0.74, 0.92; *I*^2^ = 0%; moderate-quality evidence). In population sub-group analyses, HIVST reduced linkage in FSW by 16% (RR = 0.84; 95% CI 0.75, 0.94; *I*^2^ = 19%). Results were not significant in sub-group analysis for MSM and trans people (see Fig. 6). One study provided linkage support in the form of online counselling [30], three provided a 24-h helpline [20, 23, 24] and a further two provided enhanced written information with kits [22, 27].

### Uptake and frequency of STI testing

Results related to STI testing behaviour varied. Three studies reported on this, all including MSM and one also including trans people [23, 24, 31]. One study measuring STI testing across three interventions (HIVST only, HIVST with counselling, SoC) found no differences across the three interventions [31]. A study in Australia found no differences in STI testing frequency in HIVST and SoC arms (RR = 0.92; 95% CI 0.80, 1.07) [23]. Another study in the USA delivering HIVST through facilities and mail found MSM and trans people in the HIVST arm reported significantly fewer STI tests than those in the SoC arm (HIVST arm mean = 2.3; 95% CI 1.9, 2.7; SoC arm mean = 3.2; 95% CI 2.8, 3.6; *p* = 0.0038) [24]. The first two of these studies involved facility-based distribution while the latter provided HIVST through online/mail distribution. A meta-analysis was not conducted due to heterogeneity of outcomes.

### Condom use

**Fig. 7:**
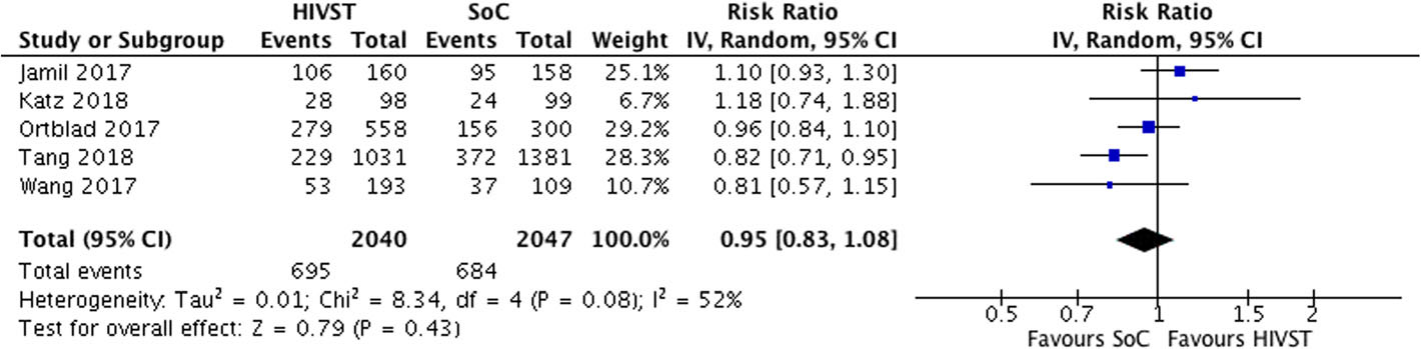
Meta-analysis of studies evaluating condomless sex among FSW, MSM and trans people

A meta-analysis of five trials found that among MSM and FSW, HIVST had no statistically significant effect on condomless sex (RR = 0.95; 95% CI 0.83, 1.08; *i*^2^ = 52%; low-quality evidence) (see Fig. 7) [23, 24, 28–30]. A further study found some evidence of difference on this outcome between HIVST interventions with and without counselling and SoC [31]. This study comparing HIVST without counselling, HIVST with counselling and SoC found that significantly fewer MSM in the HIVST and counselling arm reported CAI compared with both other interventions (IRR = 0.20, SE = 0.09, *p* < 0.001) [31]. A study of online/mail distribution found no difference in CAI partners between intervention (mean = 1.63; SD = 3.45) and SoC (mean = 1.41; SD = 2.51) arms at 12 months (*p* = 0.17) [20]. These results could not be included in the meta-analysis as they were both continuous rather than binary variables.

### Social harm or adverse events

Social harm or other adverse events were reported systematically in two RCTs among FSW, one in Uganda and one in Zambia [22, 27]. One study showed four incidents of intimate partner violence (IPV) in an HIVST arm and none in the SoC arm. Three of these events were following a partner learning of HIVST use, while the fourth was following a partner learning about engagement in sex work [22]. The other study reported two incidents of IPV in the HIVST arms and one in the SoC arm. In the HIVST arm, one person suffered verbal abuse from a boyfriend following self-testing, and the other two events were related to sex work disclosure [27]. Mental distress was reported following a positive HIVST result [27]. Neither study showed statistically significant differences across arms.

## Discussion

This systematic review and meta-analysis of 10 RCTs conducted with key populations found that distribution of free HIVST kits increases the uptake of HIV testing when compared to SoC among key populations overall and in all sub-group analyses.

HIVST increased the frequency of testing in MSM and trans people. Effect sizes for this measure were strongest in the study using an online ordering for mail distribution model. Although it did not meet our testing frequency outcome definition, and therefore was not included in our analysis, one study demonstrated that HIVST could increase the proportion of FSW who tested twice in 4 months [27].

Nine of 10 RCTs included in this review assessed the impact of HIVST on HIV positivity, with significant differences observed. Results for MSM and trans people were not significantly different among those tested, but in those randomised HIVST doubled the rate of positivity compared to SoC. Results were not significant for FSW in either analysis. This was also reflected in online/mail distribution with more infections detected in HIVST arms, while no other models showed difference. This indicates that for MSM and trans people, HIVST has the potential to increase overall yield of positive results but does not increase *test positivity* compared to standard approaches. This increase in overall number of infections detected is likely because greater numbers of MSM and trans people test when presented with HIVST compared to SoC (as demonstrated in uptake analysis) due to self-testing reducing well-documented barriers such as stigma, confidentiality concerns and issues of convenience. Online/mail delivered HIVST may have benefits over other models for similar reasons.

HIVST approaches performed less well than SoC in linking those with positive results to care overall and among FSW. Results for MSM and trans people were not statistically significantly different. It is important to note that RCTs may have been underpowered to assess these outcomes given the large number of HIV positive diagnoses required to demonstrate differences. A further issue is that many of these trials relied upon self-report for HIV positivity and linkage to care, and it may be especially challenging to confirm numbers of new positive results in an intervention which is by its nature dislocated from clinical services and potentially utilised by those with the most pronounced barriers to access. Nevertheless, careful consideration to this issue is required in HIVST implementation, especially in groups least likely to engage in care.

Evidence on the frequency of STI screening for MSM (and small numbers of trans people in one study) was mixed; two studies showed no negative effect, but one study found a significant reduction in STI testing frequency. Concerns regarding reduced STI testing with HIVST may be alleviated by providing STI self-sample testing alongside HIVST where feasible, and using a strategic approach to implement HIVST for those most likely to have unmet HIV testing need and/or undiagnosed HIV.

HIVST appears to be a safe and acceptable intervention among key populations. Encouragingly, HIVST had no negative impacts on condom use, results which counter a key concern frequently raised [6, 7, 32]. There were no serious adverse events reported by RCTs, and social harms were extremely rare, again countering common concerns [6, 7, 32, 33]. Future research on, and implementation of, HIVST for key populations should remain attentive to issues of potential harm. This is critical where individuals live in an environment of power inequalities and higher rates of background violence, salient issues for FSW, MSM and trans people [22, 27, 33, 34].

These results indicate that HIVST can be a useful approach to increase the uptake of HIV testing among MSM and FSW, with evidence of increases in testing frequency primarily available for MSM. Online ordering for mail delivery appears to have benefits over facility-based and peer distribution models, potentially because mechanisms were perceived to be more private. These differences may be due to the values and preferences of these populations regarding maximally convenient HIVST approaches requiring minimal healthcare provider interaction [6, 11, 35]. It is worth reiterating that peer/secondary distribution evidence in this review is only drawn from FSW populations, and the approach may also be useful with other populations, especially MSM and trans people. In MacGowan et al. [20], an additional person with HIV was identified through kit sharing, and this distribution approach has been used successfully in pilot and demonstration project with MSM and with trans FSW [20, 34, 36, 37]. Careful intervention design should be attentive to local contexts and the specific needs of key populations, ensuring that interventions are acceptable and accessible.

Given high acceptability [6–10, 35, 38] and effectiveness, HIVST should be implemented more widely and scaled-up among MSM. For FSW populations, although HIVST does increase testing uptake, additional caution is warranted regarding implementation because of negative impacts on linkage to care. Indeed, innovative approaches to facilitating linkage for all populations are important to optimise interventions and ensure that individuals are not lost to care.

It should be noted that heterogeneity for some outcomes was high. This is perhaps due to the inclusion of a broad range of evidence from multiple settings delivering HIVST to various populations through a multitude of intervention types. This underlines the importance of developing interventions based on the needs of the setting and the target populations.

Although our uptake funnel plot showed that overall there were few small studies all which showed benefit, we assessed this is not the result of publication bias but rather related to the inclusion of RCTs only in our review, which by their nature require sufficient numbers of patients to show effects. We assessed this as the true effect of HIVST on uptake of HIV testing, as these results mirror findings from general populations in several settings and observational studies with key populations [38–41].

### Evidence gaps

It is unlikely that many additional large-scale RCTs will be implemented. Nevertheless, some significant gaps exist in the evidence which implementation, pilot and demonstration projects can respond to.

Firstly, there was extremely limited data from trans populations, with only 72 of 9679 participants identifying as trans, no study which separated trans women from trans men and no RCT reporting outcomes for this group independently of MSM. This is a population with pronounced testing need, which observational evidence suggests HIVST may meet [8, 34].

Secondly, no RCT evidence was found for PWID or people in prisons, two groups which may also find HIVST acceptable. For people in prisons, ways to ensure confidentially and prevent coercion are critical—as they are for any testing approach in closed settings. It is also possible that these approaches will not be acceptable to staff working in these settings because of device-related safety concerns.

Finally, some outcomes of interest were not recorded in specific population groups. Adverse events were not reported in RCTs recruiting MSM and trans people (although numerous studies have shown that HIVST is highly acceptable for this group [10, 11, 35, 40, 42–46]). Condom use outcomes were collected mainly in RCTs among MSM, with only one RCT reporting on inconsistent condom use for FSW [28]. Frequency of HIV testing was only examined among MSM and a small number of trans people. Generating this evidence base and developing delivery approaches for all populations through implementation research are a critical priority.

### Strengths and limitations

This systematic review and meta-analysis has some key strengths and some important limitations. Overall, the evidence identified was of low-to-moderate quality. Many included studies relied on self-reported outcomes, including for testing uptake and positivity, which need to be taken into account when assessing evidence quality.

This is the first systematic review and meta-analysis comparing HIVST to SoC conducted exclusively using data from key populations. Our comprehensive search strategy and systematic review process is a significant strength, as is our assessment of risk of bias and independent data extraction process. Since the first meta-analysis comparing HIVST to SoC (see Johnson et al. [15]), several studies have reported which strengthen the findings, especially around uptake and frequency of testing, HIV positivity and condom use.

Our systematic approach has enabled the identification of a broad range of evidence covering MSM and FSW, with some for trans people. Further experience and evidence of HIVST for trans men and women should be prioritised as trans populations often face serious barriers to service access.

At the time of the review, there was insufficient and heterogenous evidence on differences in support tools with HIVST. When this evidence base develops further, evaluating the impact of support tools may provide additional insights into the utility of a range of supportive options which can optimise outcomes.

The vast majority of interventions in this systematic review used oral fluid HIVSTs. These have benefits in terms of ease of use but drawbacks regarding lower test sensitivity, particularly in early infection [7, 47]. Although HIVST interventions comprise multiple components in addition to the kit itself (see Table 2), this evidence base should not be considered representative of blood-based HIVSTs which require the user to collect a blood sample which is a barrier for some [6, 7, 11]. In addition, blood-based HIVSTs have improved sensitivity and may therefore identify larger numbers of HIV infections among those tested [7, 47].

A further limitation is drawn from the GRADE methodology. Allocation blinding is a central feature of risk of bias assessments within the GRADE system. Interventions like HIVST cannot be blinded, and all outcomes are therefore downgraded one position. Using this methodology which was designed with double blind trials in mind may artificially reduce our confidence in the intervention and its associated outcomes.

## Conclusions

In this review, HIVST, in RCTs, was found to be safe, and increased testing uptake, frequency and overall positivity rate for MSM and trans people when compared to standard HIV testing services, without negative effects on condom use or substantial increases in social harm. HIVST did not improve linkage to care compared to SoC in MSM and trans people. Results for female sex workers were more mixed: although testing uptake was improved, yield of positive results (test positivity or among randomised) was not and linkage outcomes were worse. Across key populations, more evidence is required to assess impact on STI testing frequency. This review highlights the importance of developing strategies to ensure linkage which meet the needs of intended beneficiaries in a variety of settings.

Among key populations, HIVST appears to engage segments of the population with pronounced barriers to standard testing services while increasing choice. HIVST therefore has an important role within broader HIV prevention efforts improving uptake of HIV testing services; it is crucial that interventions are designed in response to local need.

## Supplementary Information

**Additional file 1.** Study protocol.

**Additional file 2.** Risk of bias assessments.

**Additional file 3.** GRADE tables.

**Additional file 4.** Uptake funnel plot.

**Additional file 5.** Additional forest plots.

## Data Availability

The datasets used and/or analysed during the current study are available from the corresponding author on reasonable request.

## Footnotes

1 Two interventions reported in Wray et al. [31] were combined for this analysis as differences between them were assessed as unlikely to affect this outcome.
