# Supplementary material for: Comparing the effects of HIV self-testing to standard HIV testing for key populations: a systematic review and meta-analysis
Source: BMC Med. 2020 Dec 3;18:381. doi: 10.1186/s12916-020-01835-z (PMC7713313; doi:10.1186/s12916-020-01835-z)
Supplement: Supplementary file 1 — Additional file 1. Study protocol. [file 12916_2020_1835_MOESM1_ESM.pdf]

HIV self-testing: systematic review protocol

June 18, 2018

Background

In December 2016, WHO published the first guidelines on HIV self-testing and partner notification, a supplement to the Consolidated Guidelines on HIV testing services. These guidelines recommended that HIV self-testing (HIVST) should be offered as an additional approach to HIV testing services. Because of the limited evidence available at that time, the guidelines did not include recommendations on optimal intervention types and support tools needed to facilitate HIVST implementation. This systematic review intends to fill this gap and inform the update to the guidelines on HIV testing services. This review focuses specifically on HIV self-testing rather than community delivered or home-based HIV testing.

PICO question

Should HIV self-testing be offered as an additional approach to HIV testing services?

P: Populations receiving HIV testing services
I: HIV testing services that include self-testing
C: HIV testing services that do not include self-testing
O: Listed below

For multi-arm RCTs where all arms include HIVST:

I: HIV testing services that include HIVST and other interventions
C: HIV testing services that include HIVST only
O: Listed below

Outcomes

Outcomes to be ranked by GDG and classified into primary and secondary outcomes.

(1) *Uptake* of HIV testing services (proportion of people offered HIV testing who accepted and completed HIV testing [for self-testing proportion who accepted and completed self-testing]; in a specified time frame)
(2) *Frequency* of HIV testing i.e., number of HIV tests during a specified time frame
(3) *Social harm/adverse events* (e.g., device-related issues, coercion, relationship breakdown, violence [including intimate-partner violence, violence from family members, or community members, etc.], psycho-social harm, self-harm, economic harm, suicide, stigma, discrimination, misuse of tests)

(4) Frequency of STI screening i.e., number of STI tests during a specified time frame (either self-reported or from clinical records)
(5) Proportion of people who had a test and *diagnosed HIV-positive* (adjusted to exclude people aware of their HIV serostatus before testing and/or on antiretroviral therapy (ART))
(6) *Linkage to additional testing and clinical assessment* and/or ART initiation following a reactive self-test result (among people who have a reactive HIV test result, percentage who reach this next stage of triage, number or ART initiations)
(7) *Linkage to prevention* visit after nonreactive self-test result (among people who have a nonreactive self-test result, percentage who reach prevention services, e.g., contraception, condoms, needle and syringe programmes, opioid substitution therapy, voluntary male medical circumcision, same day ART initiation after self-testing, pre-exposure prophylaxis and post-exposure prophylaxis)
(8) *Sexual risk behavior* (e.g., condom use/condomless sex, STI diagnosis, point-of-sex testing and number of sexual partners)
(9) *Disclosure* (sharing results where beneficial to partners, support networks or family)

Inclusion criteria

To be included in the review, an article must meet the following criteria:

1) Study designs that compare people who received HTS using self-testing to people who received HTS through another modality, another model of HIVST, or to no intervention (no HTS)
2) Measured one or more of the primary or secondary outcomes listed above
3) Published in a peer-reviewed journal or conference abstract

No restrictions will be placed based on location of the intervention. No language restrictions will be used on the search. Articles in languages other than English will be translated where necessary.

Stratifications

All outcomes will be stratified and presented by the following categories:

- HIVST service delivery model: partner-delivered, facility-based, community-based, workplace, online, pharmacies/private sector, kiosks/vending machines, index partner, other
- Population type/group
  - General populations (all adults)
  - Key populations (men who have sex with men, sex workers, transgender people, persons in prisons and other closed settings and people who inject drugs)
  - Adolescents and young people (ages 10-14, 15-19, and 15-24)
- Package of services/support tool/mobilization: video support, in-person demonstrations, online counselling, financial incentives, phone call reminders, lottery

- Couples and partners, including serodiscordant couples and different types of partners (e.g. casual, steady, occasional).
- Sex/Gender: male, female, transgender, other
- Past HIV testing history/frequency (particularly for the outcome of frequency of HIV testing): last 3 months, 3-12 months, 12 months and over, never
- Education/literacy: primary, secondary, literacy etc?; high, medium, low level education
- Socio-economic status
- Income quartiles: wealthy, poverty; high-, middle- and low-income settings (world bank classifications)
- Sexual risk behavior: condomless sex, concurrent sexual partners, STI history, transactional sex, will consider other metrics

Search strategy

The following electronic databases will be searched through the search date of August 1, 2018 and then updated June 1, 2019: MEDLINE, Embase, Global Health, Social Policy and Practice, PscyINFO, Health Management Information Consortium, EBSCO CINAHL Plus, Cochrane Library and Web of Science. Secondary reference searching will also be conducted on all studies included in the review. Further, selected experts in the field will be contacted to identify additional articles not identified through other search methods. Ongoing RCTs will also be identified through search of clinical trial registries including clinicaltrials.gov, the WHO ICTRP, PACTR, and the Australian New Zealand Clinical Trials Registry.

The following conferences will also be searched for relevant conference abstracts: International AIDS Conference (IAC), International AIDS Society Conference (IAS), Interest, ASLM and Conference on Retroviruses and Opportunistic Infections (CROI). The IAC and IAS conference abstracts will be searched for all available years. For CROI, only the most recent conferences (2014 - 2018) will be searched as past conferences are inaccessible.

Search terms

The following search strategy will be adapted for all databases.

Searches will combine key terms relating to HIV (HIV, AIDS, human immune-deficiency syndrome, etc.) with terms related to self-testing (home-test, self-test, mail-test, etc.). See appendix 1 for full search strategy.

Only terms for self-testing will be used to search conference abstracts because search functions are limited.

Screening abstracts

Duplicate citations will be removed. Titles, abstracts, citation information, and descriptor terms of citations identified through the search strategy will be screened by two members of review team. Differences will be resolved by consensus, with a senior member of the research team involved if consensus cannot be reached. Full text articles will be obtained for all selected abstracts and two independent reviewers will assess all full-text articles for eligibility to determine final study selection. Differences will be resolved through consensus.

Citations identified will be initially screened into the following categories:

- **Include on title and abstract** – Used when the article appears to meet the inclusion criteria.
- **Full text screen** – Used when the article may or may not meet the inclusion criteria, and the full text of the article must be reviewed before final decision about inclusion can be made.
- **Include on full text screen** – Used when the article appears to meet the inclusion criteria for the primary review on full text screening.
- **No** – Used when the article clearly does not meet the inclusion criteria for the review and no further consideration is necessary.

Data extraction and management

Data will be extracted independently by two reviewers using standardized data extraction forms. Differences in data extraction will be resolved through consensus or referral to a senior study team member when consensus cannot be reached.

The following information will be extracted from each included study:

- Study identification: Author(s); type of citation; year of publication
- Study description: Study objectives; location; population characteristics; description of the type of HIV testing (for both self-testing and other testing arms); description of any additional intervention components; study design; sample size; follow-up periods and loss to follow-up
- Outcomes: Analytic approach; outcome measures; comparison groups; effect sizes; confidence intervals; significance levels; conclusions; limitations

Quality assessment

For randomized trials, risk of bias will be assessed by two members of the study team using the Cochrane Collaboration's tool for assessing risk of bias (Cochrane Handbook, chapter 8.5 – Higgins & Green, 2011). This tool assesses random sequence generation (selection bias), allocation concealment (selection bias), blinding of participants and personnel (performance bias), blinding of outcome assessment (detection bias) blinding of outcome assessment (detection bias), incomplete outcome data addressed (attrition bias), incomplete outcome data,

and selective reporting (reporting bias). Methodological components of the studies will be assessed and classified as high or low risk of bias. For non-randomized trials but comparative studies, study rigor will be assessed using the Evidence Project 8-item checklist for intervention evaluations.

Grading of evidence

Following the GRADE approach, if direct evidence for this PICO question is limited or non-existent, indirect evidence will be used instead, but downgraded for indirectness. Similarly, if evidence from randomized controlled trials is limited, evidence from non-randomized but controlled studies will be used instead, but also downgraded per the GRADE system.

Data analysis

Data will be analyzed according to coding categories and outcomes. Where there are multiple studies reporting the same outcome, meta-analysis will be conducted using random-effects models to combine risk ratios using the program Comprehensive Meta-Analysis (CMA). For studies with continuous data, meta-analysis will use random effects models to combine standardised mean difference. Rate ratios will be used for frequency data. Data will be summarized in GRADE tables, summary of finding tables, and risk/benefit tables.

Values and preferences review

Studies that include outcomes of interest but are non-randomised or without a control group and where participants performed self-testing will be included in this review. Studies will be included if they present primary data, qualitative or quantitative, examining people's accounts of peoples' acceptability and values and preferences regarding self-testing. Values and preferences data will be summarized qualitatively and will be organized by study design and methodology, location, and population.

Cost and resource needs

Information related to cost and cost-effectiveness of HIV self-testing will be extracted from randomized studies included in the review, where available. Costs and resources may include those on staffing, training, compensation, equipment (including test kits), and infrastructure. Such information will be summarized and reported qualitatively. This information will only be included if based on actual or programme costing data, modelled cost analyses will not be included.

References

Higgins, J.P.T., & Green, S. (2011). *Cochrane Handbook for Systematic Reviews of Interventions.* Version 5.1.0. Available from: http://www.cochrane-handbook.org/. Accessed on: June 10, 2011.

Appendix 1: search strategy

The following databases were searched:

- Ovid SP Medline (Epub Ahead of Print, In-Process & Other Non-Indexed Citations, Ovid MEDLINE(R) Daily and Ovid MEDLINE(R) 1946 to Present, searched on 14 October 2016
- OvidSP Embase 1974 to 2016 October 13, searched on 14 October 2016
- OvidSP Global Health 1910 to 2016 week 39, searched on 14 October 2016
- OvidSP Social Policy & Practice 201607, searched on 14 October 2016
- OvidSP PsycINFO 1806 to October week 1 2016, searched on 14 October 2016
- OvidSP HMIC 1979 to September 2016, searched on 14 October 2016
- OvidSP Econlit 1886 to September 2016, searched on 18 October 2016
- EBSCOhost CINAHL Plus, searched on 14 October 2016
- Wiley Cochrane Library, searched on 14 October 2016
- Thomson Reuters Web of Science Core Collection, searched on 17 October 2016
- Elsevier SCOPUS, searched on 17 October 2016

All searched were limited to English language only and to results published from 2006 to present. Complete search strategies for each database are provided below. A total of 8860 items were retrieved. After duplicates were removed, 3571 unique items were retrieved.

For each database, these searches were used to create a monthly alert which emailed new papers to Charles Witzel.

Search strategies used

Database: OvidSP MEDLINE

Numbers in brackets are the number of items retrieved.

1. exp HIV Infections/ (253181)
2. exp HIV/ (89960)
3. hiv.ti,ab. (268385)
4. "hiv1".ti,ab. (793)
5. "hiv2".ti,ab. (155)
6. “hiv type 1".ti,ab. (3775)
7. "hiv type 2".ti,ab. (182)
8. human immunodeficiency virus.ti,ab. (77583)
9. human immunedeficiency virus.ti,ab. (6)
10. human immuno-deficiency virus.ti,ab. (214)
11. human immune-deficiency virus.ti,ab. (304)
12. (human immun* adj3 deficiency virus).ti,ab. (523)
13. acquired immunodeficiency syndrome.ti,ab. (15302)
14. acquired immunedeficiency syndrome.ti,ab. (13)
15. acquired immuno-deficiency syndrome.ti,ab. (116)
16. acquired immune-deficiency syndrome.ti,ab. (5387)
17. (acquired immun* adj3 deficiency syndrome).ti,ab. (5542)
18. Sexually Transmitted Diseases, Viral/ (1313)
19. or/1-18 (358848)
20. 20 (sample adj1 collect*).ti,ab. (8260)
21. Dried Blood Spot Testing/ and (home* or remote or personal or self*).ti,ab. (49)
22. (dried blood spot adj3 (home* or remote or personal or self*)).ti,ab. (16)

23. (alternative adj3 test*).ti,ab. (4973)
24. (option* adj1 test*).ti,ab. (408)
25. ((Home* or self* or mail*) adj3 (collection* or sampl* or specimen* or test* or kit)).ti,ab. (14504)
26. or/20-25 (27987)
27. 19 and 26 (1399)
28. limit 27 to english language (1357)
29. limit 28 to yr="2006 -Current" (911)

Database: OvidSP Embase

Numbers in brackets are the number of items retrieved

1. exp Human immunodeficiency virus/ (236134)
2. exp Human immunodeficiency virus infection/ (338249)
3. hiv.ti,ab. (318347)
4. "hiv1".ti,ab. (1244)
5. "hiv2".ti,ab. (230)
6. "hiv type 1".ti,ab. (3840)
7. "hiv type 2".ti,ab. (188)
8. human immunodeficiency virus.ti,ab. (81710)
9. human immunedeficiency virus.ti,ab. (24)
10. human immuno-deficiency virus.ti,ab. (258)
11. human immune-deficiency virus.ti,ab. (381)
12. (human immun* adj3 deficiency virus).ti,ab. (646)
13. acquired immunodeficiency syndrome.ti,ab. (15678)
14. acquired immunedeficiency syndrome.ti,ab. (22)
15. acquired immuno-deficiency syndrome.ti,ab. (136)
16. acquired immune-deficiency syndrome.ti,ab. (5665)
17. (acquired immun* adj3 deficiency syndrome).ti,ab. (5828)
18. or/1-17 (460612)
19. (sample adj1 collect*).ti,ab. (11106)
20. Dried Blood Spot Testing/ and (home* or remote or personal or self*).ti,ab. (214)
21. (dried blood spot adj3 (home* or remote or personal or self*)).ti,ab. (21)
22. (alternative adj3 test*).ti,ab. (6145)
23. (option* adj1 test*).ti,ab. (569)
24. ((Home* or self* or mail*) adj3 (collection* or sampl* or specimen* or test* or kit)).ti,ab. (18541)
25. or/19-24 (36241)
26. 18 and 25 (1838)
27. limit 26 to english language (1789)
28. limit 27 to yr="2006 -Current" (1337)

Database: OvidSP Global Health

Numbers in brackets are the number of items retrieved

1. exp human immunodeficiency viruses/ (151887)
2. hiv.ti,ab. (129335)
3. "hiv1".ti,ab. (252)
4. "hiv2".ti,ab. (36)
5. "hiv type 1".ti,ab. (2139)
6. "hiv type 2".ti,ab. (98)
7. human immunodeficiency virus.ti,ab. (36609)

8. human immunedeficiency virus.ti,ab. (3)
9. human immuno-deficiency virus.ti,ab. (71)
10. human immune-deficiency virus.ti,ab. (139)
11. (human immun* adj3 deficiency virus).ti,ab. (210)
12. acquired immunodeficiency syndrome.ti,ab. (4538)
13. acquired immunedeficiency syndrome.ti,ab. (4)
14. acquired immuno-deficiency syndrome.ti,ab. (39)
15. acquired immune-deficiency syndrome.ti,ab. (3398)
16. (acquired immun* adj3 deficiency syndrome).ti,ab. (3443)
17. or/2-17 (163755)
18. (sample adj1 collect*).ti,ab. (2272)
19. (dried blood spot adj3 (home* or remote or personal or self*)).ti,ab. (6)
20. (alternative adj3 test*).ti,ab. (851)
21. (option* adj1 test*).ti,ab. (83)
22. ((Home* or self* or mail*) adj3 (collection* or sampl* or specimen* or test* or kit)).ti,ab. (3760)
23. or/19-23 (6873)
24. 18 and 24 (749)
25. limit 25 to english language (715)
26. limit 26 to yr="2006 -Current" (563)

Database: OvidSP Social Policy and Practice

Numbers in brackets are the number of items retrieved

1. hiv.ti,ab. (4096)
2. "hiv1".ti,ab. (2)
3. "hiv2".ti,ab. (0)
4. "hiv type 1".ti,ab. (1)
5. "hiv type 2".ti,ab. (0)
6. human immunodeficiency virus.ti,ab. (68)
7. human immunedeficiency virus.ti,ab. (0)
8. human immuno-deficiency virus.ti,ab. (4)
9. human immune-deficiency virus.ti,ab. (2)
10. (human immun* adj3 deficiency virus).ti,ab. (6)
11. acquired immunodeficiency syndrome.ti,ab. (19)
12. acquired immunedeficiency syndrome.ti,ab. (0)
13. acquired immuno-deficiency syndrome.ti,ab. (0)
14. acquired immune-deficiency syndrome.ti,ab. (19)
15. (acquired immun* adj3 deficiency syndrome).ti,ab. (19)
16. or/1-15 (4116)
17. (sample adj1 collect*).ti,ab. (8)
18. (dried blood spot adj3 (home* or remote or personal or self*)).ti,ab. (0)
19. (alternative adj3 test*).ti,ab. (24)
20. (option* adj1 test*).ti,ab. (3)
21. ((Home* or self* or mail*) adj3 (collection* or sampl* or specimen* or test* or kit)).ti,ab. (455)
22. or/17-21 (488)
23. 16 and 22 (11)

Database: OvidSP PsycINFO

Numbers in brackets are the number of items retrieved

1. HIV testing/ (1916)
2. exp HIV/ (36936)
3. hiv.ti,ab. (42881)
4. "hiv1".ti,ab. (22)
5. "hiv2".ti,ab. (6)
6. "hiv type 1".ti,ab. (57)
7. "hiv type 2".ti,ab. (1)
8. human immunodeficiency virus.ti,ab. (5093)
9. human immunedeficiency virus.ti,ab. (1)
10. human immuno-deficiency virus.ti,ab. (12)
11. human immune-deficiency virus.ti,ab. (25)
12. (human immun* adj3 deficiency virus).ti,ab. (38)
13. acquired immunodeficiency syndrome.ti,ab. (669)
14. acquired immunedeficiency syndrome.ti,ab. (0)
15. acquired immuno-deficiency syndrome.ti,ab. (4)
16. acquired immune-deficiency syndrome.ti,ab. (2866)
17. (acquired immun* adj3 deficiency syndrome).ti,ab. (2873)
18. or/1-17 (47745)
19. (sample adj1 collect*).ti,ab. (436)
20. (dried blood spot adj3 (home* or remote or personal or self*)).ti,ab. (3)
21. (alternative adj3 test*).ti,ab. (1972)
22. (option* adj1 test*).ti,ab. (108)
23. ((Home* or self* or mail*) adj3 (collection* or sampl* or specimen* or test* or kit)).ti,ab. (10008)
24. or/19-23 (12484)
25. 18 and 24 (382)
26. limit 25 to english language (367)
27. limit 26 to yr="2006 -Current" (270)

Database: Ovid SP HMIC Health Management Information Consortium

Numbers in brackets are the number of items retrieved

1. hiv tests/ (108)
2. HIV/ (3490)
3. acquired immune deficiency syndrome/ (3178)
4. hiv.ti,ab. (4302)
5. "hiv1".ti,ab. (2)
6. "hiv2".ti,ab. (0)
7. "hiv type 1".ti,ab. (0)
8. "hiv type 2".ti,ab. (0)
9. human immunodeficiency virus.ti,ab. (607)
10. human immunedeficiency virus.ti,ab. (0)
11. human immuno-deficiency virus.ti,ab. (4)
12. human immune-deficiency virus.ti,ab. (24)
13. (human immun* adj3 deficiency virus).ti,ab. (28)
14. acquired immunodeficiency syndrome.ti,ab. (114)
15. acquired immunedeficiency syndrome.ti,ab. (0)
16. acquired immuno-deficiency syndrome.ti,ab. (1)
17. acquired immune-deficiency syndrome.ti,ab. (181)
18. (acquired immun* adj3 deficiency syndrome).ti,ab. (182)

19. or/1-18 (6027)
20. (sample adj1 collect*).ti,ab. (13)
21. (dried blood spot adj3 (home* or remote or personal or self*)).ti,ab. (0)
22. (alternative adj3 test*).ti,ab. (41)
23. (option* adj1 test*).ti,ab. (9)
24. ((Home* or self* or mail*) adj3 (collection* or sampl* or specimen* or test* or kit)).ti,ab. (375)
25. or/20-24 (438)
26. 19 and 25 (17)
27. limit 26 to yr="2006 -Current" (9)

Database: OvidSP EconLit
Numbers in brackets are the number of items retrieved
1. hiv.ti,ab. (1630)
2. "hiv1".ti,ab. (0)
3. "hiv2".ti,ab. (0)
4. "hiv type 1".ti,ab. (0)
5. "hiv type 2".ti,ab. (0)
6. human immunodeficiency virus.ti,ab. (70)
7. human immunedeficiency virus.ti,ab. (0)
8. human immuno-deficiency virus.ti,ab. (0)
9. human immune-deficiency virus.ti,ab. (0)
10. (human immun* adj3 deficiency virus).ti,ab. (0)
11. acquired immunodeficiency syndrome.ti,ab. (8)
12. acquired immunedeficiency syndrome.ti,ab. (0)
13. acquired immuno-deficiency syndrome.ti,ab. (2)
14. acquired immune-deficiency syndrome.ti,ab. (24)
15. (acquired immun* adj3 deficiency syndrome).ti,ab. (26)
16. or/1-15 (1664)
17. (sample adj1 collect*).ti,ab. (59)
18. (dried blood spot adj3 (home* or remote or personal or self*)).ti,ab. (0)
19. (alternative adj3 test*).ti,ab. (1065)
20. (option* adj1 test*).ti,ab. (17)
21. ((Home* or self* or mail*) adj3 (collection* or sampl* or specimen* or test* or kit)).ti,ab. (398)
22. or/17-21 (1538)
23. 16 and 22 (2)

Database: EBSCO CINAHL Plus with Full Text
Numbers in brackets are the number of items retrieved
S1. (MH "HIV Infections+") (67,057)
S2. (MH "Human Immunodeficiency Virus+") (6,368)
S3. (TI hiv) OR (AB hiv) (61,304)
S4. (TI hiv1) OR (AB hiv1) (3,838)
S5. (TI hiv2) or (AB hiv2) (175)
S6. (TI hiv type 1) or (AB hiv type 1) (624)
S7. (TI hiv type 2) or (AB hiv type 2) (13)
S8. (TI human immunodeficiency virus) or (AB human immunodeficiency virus) (35,129)
S9. (TI human immunedeficiency virus) OR (AB human immunedeficiency virus) (0)
S10. (TI human immuno-deficiency virus) or (AB human immuno-deficiency virus) (19)

S11. (TI human immune-deficiency virus) OR (AB human immune-deficiency virus) (30)
S12. human immun* N3 deficiency virus (49)
S13. (TI acquired immunodeficiency syndrome) or (AB acquired immunodeficiency syndrome) (1,911)
S14. (TI acquired immunedeficiency syndrome) or (AB acquired immunedeficiency syndrome) (1)
S15. (TI acquired immuno-deficiency syndrome) OR (AB acquired immuno-deficiency syndrome) (1,655)
S16. (TI acquired immune-deficiency syndrome) or (AB acquired immune-deficiency syndrome) (1,785)
S17. acquired immun* N3 deficiency syndrome (662)
S18. S1 OR S2 OR S3 OR S4 OR S5 OR S6 OR S7 OR S8 OR S9 OR S10 OR S11 OR S12 OR S13 OR S14 OR S15 OR S16 OR S17 (85,288)
S19. (MH "Home Diagnostic Tests") (511)
S20. sample N1 collect* (6,919)
S21. (TI dried blood spot N3 (home* or remote or personal or self*)) or (AB dried blood spot N3 (home* or remote or personal or self*)) (7)
S22. alternative N3 test* (777)
S23. option* N1 test* (182)
S24. (home* N3 collection*) OR (home* N3 sampl*) or (home* N3 specimen*) or (home* N3 test*) or (home* N3 kit) or (self* N3 collection*) or (self* N3 sampl*) or (self* N3 specimen*) or (self* N3 test*) or (self* N3 kit) or (mail* N3 collection*) or (mail* N3 sampl*) or (mail* N3 specimen*) or (mail* N3 test*) or (mail* N3 kit) (7,399)
S25. S19 OR S20 OR S21 OR S22 OR S23 OR S24 (15,096)
S26. S18 AND S25 (766)
S27. S26 Limiters – English Language (762)
S28. S27 Limiters – Published date: 20060101 – (561)

Database: Cochrane Library

Numbers in brackets are the number of items retrieved

#1. MeSH descriptor: [HIV Infections] explode all trees (9156)
#2. MeSH descriptor: [HIV] explode all trees (2885)
#3. hiv:ti,ab,kw (Word variations have been searched) (14031)
#4. "hiv1":ti,ab,kw (Word variations have been searched) (14)
#5. "hiv2":ti,ab,kw (Word variations have been searched) (0)
#6. "hiv type 1":ti,ab,kw (Word variations have been searched) (191)
#7. "hiv type 2":ti,ab,kw (Word variations have been searched) (0)
#8. "human immunodeficiency virus":ti,ab,kw (Word variations have been searched) (5774)
#9. "human immunedeficiency virus":ti,ab,kw (Word variations have been searched) (0)
#10. "human immuno-deficiency virus":ti,ab,kw (Word variations have been searched) (9)
#11. "human immune-deficiency virus":ti,ab,kw (Word variations have been searched) (3)
#12. "human immun*" near/3 "deficiency virus":ti,ab,kw (Word variations have been searched) (12)
#13. "acquired immunodeficiency syndrome":ti,ab,kw (Word variations have been searched) (1494)
#14. "acquired immunedeficiency syndrome":ti,ab,kw (Word variations have been searched) (1)
#15. "acquired immuno-deficiency syndrome":ti,ab,kw (Word variations have been searched) (2)
#16. "acquired immune-deficiency syndrome":ti,ab,kw (Word variations have been searched) (562)
#17. "acquired immun*" near/3 "deficiency syndrome":ti,ab,kw (Word variations have been searched) (566)
#18. MeSH descriptor: [Sexually Transmitted Diseases, Viral] this term only (25)

#19. #1 or #2 or #3 or #4 or #5 or #6 or #7 or #8 or #9 or #10 or #11 or #12 or #13 or #14 or #15 or #16 or #17 or #18 (15086)
#20. sample near/1 collect*:ti,ab,kw (Word variations have been searched) (1438)
#21. ("dried blood spot" near/3 home*) or ("dried blood spot" near/3 remote) or ("dried blood spot" near/3 personal) or ("dried blood spot" near/3 self*):ti,ab,kw (Word variations have been searched) (2)
#22. alternative near/3 test*:ti,ab,kw (Word variations have been searched) (257)
#23. option* near/1 test*:ti,ab,kw (Word variations have been searched) (20)
#24. (home* near/3 collection*) or (home* near/3 sampl*) or (home* near/3 specimen*) or (home* near/3 test*) or (home* near/3 kit) or (self* near/3 collection*) or (self* near/3 sampl*) or (self* near/3 specimen*) or (self* near/3 test*) or (self* near/3 kit) or (mail* near/3 collection*) or (mail* near/3 sampl*) or (mail* near/3 specimen*) or (mail* near/3 test*) or (mail* near/3 kit):ti,ab,kw (Word variations have been searched) (2503)
#25. #20 or #21 or #22 or #23 or #24 (4161)
#26. #19 and #25 (216)
This breaks down as:

- Cochrane Reviews (8)
- Other Reviews (1)
- Trials (201)
- Methods Studies (6)
- Technology Assessments (0)
- Economic Evaluations (0)
- Cochrane Groups (0)

Database: Web of Science™ Core Collection
Numbers in brackets are the number of items retrieved. All searches run across Indexes = SCI-EXPANDED, SSCI, A&HCI, CPCI-S, CPCI-SSH, ESCI. Timespan = 2006-2016
#1. TOPIC: (hiv) (162,466)
#2. TOPIC: (hiv1) (338)
#3. TOPIC: (hiv2) (32)
#4. TOPIC: ("hiv type 1") (1,561)
#5. TOPIC: ("hiv type 2") (50)
#6. TOPIC: ("human immunodeficiency virus") (43,584)
#7. TOPIC: ("human immunedeficiency virus") (4)
#8. TOPIC: ("human immuno-deficiency virus") (80)
#9. TOPIC: ("human immune-deficiency virus") (119)
#10. TOPIC: ("human immun*" NEAR/3 "deficiency virus") (202)
#11. TOPIC: ("acquired immunodeficiency syndrome") (4,393)
#12. TOPIC: ("acquired immunedeficiency syndrome") (1)
#13. TOPIC: ("acquired immuno-deficiency syndrome") (31)
#14. TOPIC: ("acquired immune-deficiency syndrome") (1,046)
#15. #14 OR #13 OR #12 OR #11 OR #10 OR #9 OR #8 OR #7 OR #6 OR #5 OR #4 OR #3 OR #2 OR #1 (170,925)
#16. TOPIC: (sample NEAR/1 collect*) (93,366)
#17. TOPIC: ("dried blood spot" NEAR/3 home*) (10)
#18. TOPIC: ("dried blood spot" NEAR/3 remote) (1)
#19. TOPIC: ("dried blood spot" NEAR/3 personal) (2)

#20. TOPIC: ("dried blood spot" NEAR/3 self*) (7)
#21. TOPIC: (alternative NEAR/3 test*) (7,486)
#22. TOPIC: (option* NEAR/1 test*) (710)
#23. TOPIC: (home* NEAR/3 collection*) (308)
#24. TOPIC: (home* NEAR/3 sampl*) (1,878)
#25. TOPIC: (home* NEAR/3 specimen*) (66)
#26. TOPIC: (home* NEAR/3 test*) (2,607)
#27. TOPIC: (home* NEAR/3 kit) (198)
#28. TOPIC: (self* NEAR/3 collection*) (652)
#29. TOPIC: (self* NEAR/3 sampl*) (3,407)
#30. TOPIC: (self* NEAR/3 specimen*) (405)
#31. TOPIC: (self* NEAR/3 test*) (8,991)
#32. TOPIC: (self* NEAR/3 kit) (200)
#33. TOPIC: (mail* NEAR/3 collection*) (79)
#34. TOPIC: (mail* NEAR/3 sampl*) (577)
#35. TOPIC: (mail* NEAR/3 specimen*) (25)
#36. TOPIC: (mail* NEAR/3 test*) (227)
#37. TOPIC: (mail* NEAR/3 kit) (81)
#38. #37 OR #36 OR #35 OR #34 OR #33 OR #32 OR #31 OR #30 OR #29 OR #28 OR #27 OR #26 OR #25 OR #24 OR #23 OR #22 OR #21 OR #20 OR #19 OR #18 OR #17 OR #16 (119,346)
#39. #38 AND #15 (2,427)
#40. #38 AND #15 Refined by: LANGUAGES: (ENGLISH) (2,393)

Database: SCOPUS

Numbers in brackets are the number of items retrieved

((TITLE-ABS-KEY (hiv)) OR (TITLE-ABS-KEY ("hiv1")) OR (TITLE-ABS-KEY ("hiv2")) OR (TITLE-ABS-KEY ("hiv type 1")) OR (TITLE-ABS-KEY ("hiv type 2")) OR (TITLE-ABS-KEY ("human immunodeficiency virus")) OR (TITLE-ABS-KEY ("human immunedeficiency virus")) OR (TITLE-ABS-KEY ("human immuno-deficiency virus")) OR (TITLE-ABS-KEY ("human immune-deficiency virus")) OR (TITLE-ABS-KEY ("human immun*" W/3 "deficiency virus")) OR (TITLE-ABS-KEY ("acquired immunodeficiency syndrome")) OR (TITLE-ABS-KEY ("acquired immunedeficiency syndrome")) OR (TITLE-ABS-KEY ("acquired immuno-deficiency syndrome")) OR (TITLE-ABS-KEY ("acquired immune-deficiency syndrome")) OR (TITLE-ABS-KEY ("acquired immun*" W/3 "deficiency syndrome"))) AND ((TITLE-ABS-KEY (sample W/1 collect*)) OR (TITLE-ABS-KEY ("dried blood spot" W/3 home*)) OR (TITLE-ABS-KEY ("dried blood spot" W/3 remote)) OR (TITLE-ABS-KEY ("dried blood spot" W/3 personal)) OR (TITLE-ABS-KEY ("dried blood spot" W/3 self*)) OR (TITLE-ABS-KEY (alternative W/3 test*)) OR (TITLE-ABS-KEY (option W/1 test*)) OR (TITLE-ABS-KEY (home* W/3 collection*)) OR (TITLE-ABS-KEY (home* W/3 sampl*)) OR (TITLE-ABS-KEY (home* W/3 specimen*)) OR (TITLE-ABS-KEY (home* W/3 test*)) OR (TITLE-ABS-KEY (home* W/3 kit)) OR (TITLE-ABS-KEY (self* W/3 collection*)) OR (TITLE-ABS-KEY (self* W/3 sampl*)) OR (TITLE-ABS-KEY (self* W/3 specimen*)) OR (TITLE-ABS-KEY (self* W/3 test*)) OR (TITLE-ABS-KEY (self* W/3 kit)) OR (TITLE-ABS-KEY (mail* W/3 collection*)) OR (TITLE-ABS-KEY (mail* W/3 sampl*)) OR (TITLE-ABS-KEY (mail* W/3 specimen*)) OR (TITLE-ABS-KEY (mail* W/3 test*)) OR (TITLE-ABS-KEY (mail* W/3 kit))) AND (LIMIT-TO (PUBYEAR , 2017) OR LIMIT-TO (PUBYEAR , 2016) OR LIMIT-TO (PUBYEAR , 2015) OR LIMIT-TO (PUBYEAR , 2014) OR LIMIT-TO (PUBYEAR , 2013) OR LIMIT-TO (PUBYEAR , 2012) OR LIMIT-TO (PUBYEAR , 2011) OR LIMIT-TO (PUBYEAR , 2010) OR LIMIT-TO (PUBYEAR ,

2009) OR LIMIT-TO (PUBYEAR , 2008) OR LIMIT-TO (PUBYEAR , 2007) OR LIMIT-TO (PUBYEAR , 2006)) AND (LIMIT-TO (LANGUAGE , "English")) (2587)
