# Supplementary material for: Comparing the effects of HIV self-testing to standard HIV testing for key populations: a systematic review and meta-analysis
Source: BMC Med. 2020 Dec 3;18:381. doi: 10.1186/s12916-020-01835-z (PMC7713313; doi:10.1186/s12916-020-01835-z)
Supplement: Supplementary file 2 — Additional file 2. Risk of bias assessments. [file 12916_2020_1835_MOESM2_ESM.docx]

**Uptake of HIV testing**

| **Study** | **Random sequence generation (selection bias)** | **Allocation concealment (selection bias)** | **Blinding of participants and personnel (performance bias)** | **Blinding of outcome assessment (detection bias)** | **Incomplete outcome data (attrition bias)** | **Selective reporting (reporting bias)** | **Other bias** | **Recruitment bias** | **Cluster imbalance** | **Loss of cluster** | **Incorrect analysis** | **Overall risk of bias** |
| --- | --- | --- | --- | --- | --- | --- | --- | --- | --- | --- | --- | --- |
|  | **ROB domains** | | | | | | | **cROB domains** | | | |  |
| **Chanda 2017** | 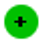 | 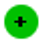 | 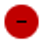 | 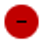 | 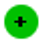 | 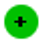 | 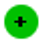 | 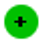 | 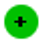 | 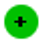 | 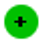 | 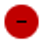 |
| **Jamil 2017** | 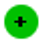 | 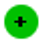 | 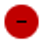 | 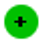 | 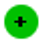 | 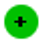 | 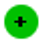 |  |  |  |  | 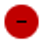 |
| **Katz 2018** | 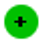 | 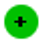 | 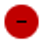 | 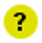 | 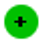 | 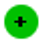 | 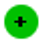 |  |  |  |  | 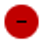 |
| **Kelvin 2019b** | 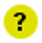 | 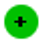 | 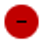 | 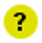 | 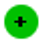 | 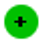 | 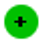 |  |  |  |  | 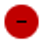 |
| **MacGowan 2017** | 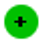 | 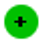 | 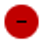 | 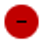 | 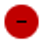 | 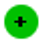 | 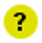 |  |  |  |  | 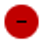 |
| **Masters 2016** | 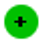 | 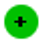 | 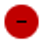 | 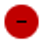 | 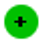 | 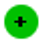 | 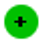 |  |  |  |  | 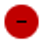 |
| **Ortblad 2017** | 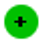 | 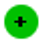 | 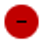 | 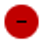 | 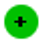 | 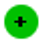 | 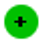 | 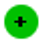 | 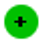 | 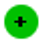 | 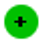 | 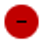 |
| **Tang 2018** | 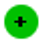 | 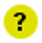 | 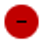 | 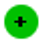 | 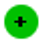 | 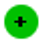 | 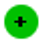 | 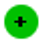 | 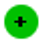 | 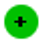 | 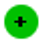 | 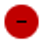 |
| **Wang 2017** | 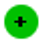 | 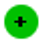 | 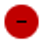 | 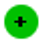 | 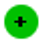 | 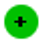 | 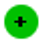 |  |  |  |  | 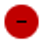 |
| **Wray 2018** | 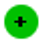 | 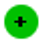 | 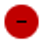 | 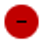 | 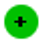 | 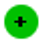 | 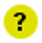 |  |  |  |  | 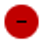 |

**Frequency of HIV testing**

| **Study** | **Random sequence generation (selection bias)** | **Allocation concealment (selection bias)** | **Blinding of participants and personnel (performance bias)** | **Blinding of outcome assessment (detection bias)** | **Incomplete outcome data (attrition bias)** | **Selective reporting (reporting bias)** | **Other bias** | **Recruitment bias** | **Cluster imbalance** | **Loss of cluster** | **Incorrect analysis** | **Overall risk of bias** |
| --- | --- | --- | --- | --- | --- | --- | --- | --- | --- | --- | --- | --- |
|  | **ROB domains** | | | | | | | **cROB domains** | | | |  |
| **Jamil 2017** | 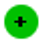 | 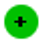 | 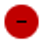 | 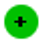 | 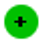 | 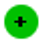 | 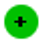 |  |  |  |  | 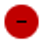 |
| **Katz 2018** |  |  |  |  |  |  |  |  |  |  |  |  |
| **MacGowan 2017** |  |  |  |  |  |  |  |  |  |  |  |  |

**HIV positivity rate**

| **Study** | **Random sequence generation (selection bias)** | **Allocation concealment (selection bias)** | **Blinding of participants and personnel (performance bias)** | **Blinding of outcome assessment (detection bias)** | **Incomplete outcome data (attrition bias)** | **Selective reporting (reporting bias)** | **Other bias** | **Recruitment bias** | **Cluster imbalance** | **Loss of cluster** | **Incorrect analysis** | **Overall risk of bias** |
| --- | --- | --- | --- | --- | --- | --- | --- | --- | --- | --- | --- | --- |
|  | **ROB domains** | | | | | | | **cROB domains** | | | |  |
| **Chanda 2017** |  |  |  |  |  |  |  |  |  |  |  |  |
| **Jamil 2017** |  |  |  |  |  |  |  |  |  |  |  |  |
| **Katz 2018** |  |  |  |  |  |  |  |  |  |  |  |  |
| **Kelvin 2019b** |  |  |  |  |  |  |  |  |  |  |  |  |
| **MacGowan 2017** |  |  |  |  |  |  |  |  |  |  |  |  |
| **Merchant 2018** |  |  |  |  |  |  |  |  |  |  |  |  |
| **Ortblad 2017** |  |  |  |  |  |  |  |  |  |  |  |  |
| **Wang 2017** |  |  |  |  |  |  |  |  |  |  |  |  |
| **Wray 2018** |  |  |  |  |  |  |  |  |  |  |  |  |

**Linkage to HIV care or ART initiation**

| **Study** | **Random sequence generation (selection bias)** | **Allocation concealment (selection bias)** | **Blinding of participants and personnel (performance bias)** | **Blinding of outcome assessment (detection bias)** | **Incomplete outcome data (attrition bias)** | **Selective reporting (reporting bias)** | **Other bias** | **Recruitment bias** | **Cluster imbalance** | **Loss of cluster** | **Incorrect analysis** | **Overall risk of bias** |
| --- | --- | --- | --- | --- | --- | --- | --- | --- | --- | --- | --- | --- |
|  | **ROB domains** | | | | | | | **cROB domains** | | | |  |
| **Chanda 2017** |  |  |  |  |  |  |  |  |  |  |  |  |
| **Jamil 2017** |  |  |  |  |  |  |  |  |  |  |  |  |
| **Katz 2018** |  |  |  |  |  |  |  |  |  |  |  |  |
| **MacGowan 2019** |  |  |  |  |  |  |  |  |  |  |  |  |
| **Ortblad 2017** |  |  |  |  |  |  |  |  |  |  |  |  |
| **Wang 2017** |  |  |  |  |  |  |  |  |  |  |  |  |

**Frequency of STI testing**

| **Study** | **Random sequence generation (selection bias)** | **Allocation concealment (selection bias)** | **Blinding of participants and personnel (performance bias)** | **Blinding of outcome assessment (detection bias)** | **Incomplete outcome data (attrition bias)** | **Selective reporting (reporting bias)** | **Other bias** | **Recruitment bias** | **Cluster imbalance** | **Loss of cluster** | **Incorrect analysis** | **Overall risk of bias** |
| --- | --- | --- | --- | --- | --- | --- | --- | --- | --- | --- | --- | --- |
|  | **ROB domains** | | | | | | | **cROB domains** | | | |  |
| **Jamil 2017** |  |  |  |  |  |  |  |  |  |  |  |  |
| **Katz 2018** |  |  |  |  |  |  |  |  |  |  |  |  |
| **Wray 2018** |  |  |  |  |  |  |  |  |  |  |  |  |

**Social harms or adverse events**

| **Study** | **Random sequence generation (selection bias)** | **Allocation concealment (selection bias)** | **Blinding of participants and personnel (performance bias)** | **Blinding of outcome assessment (detection bias)** | **Incomplete outcome data (attrition bias)** | **Selective reporting (reporting bias)** | **Other bias** | **Recruitment bias** | **Cluster imbalance** | **Loss of cluster** | **Incorrect analysis** | **Overall risk of bias** |
| --- | --- | --- | --- | --- | --- | --- | --- | --- | --- | --- | --- | --- |
|  | **ROB domains** | | | | | | | **cROB domains** | | | |  |
| **Chanda 2017** |  |  |  |  |  |  |  |  |  |  |  |  |
| **Ortblad 2017** |  |  |  |  |  |  |  |  |  |  |  |  |

**Condom use or condomless sex**

| **Study** | **Random sequence generation (selection bias)** | **Allocation concealment (selection bias)** | **Blinding of participants and personnel (performance bias)** | **Blinding of outcome assessment (detection bias)** | **Incomplete outcome data (attrition bias)** | **Selective reporting (reporting bias)** | **Other bias** | **Recruitment bias** | **Cluster imbalance** | **Loss of cluster** | **Incorrect analysis** | **Overall risk of bias** |
| --- | --- | --- | --- | --- | --- | --- | --- | --- | --- | --- | --- | --- |
|  | **ROB domains** | | | | | | | **cROB domains** | | | |  |
| **Jamil 2017** |  |  |  |  |  |  |  |  |  |  |  |  |
| **Katz 2018** |  |  |  |  |  |  |  |  |  |  |  |  |
| **MacGowan 2019** |  |  |  |  |  |  |  |  |  |  |  |  |
| **Ortblad 2017** |  |  |  |  |  |  |  |  |  |  |  |  |
| **Tang 2018** |  |  |  |  |  |  |  |  |  |  |  |  |
| **Wang 2017** |  |  |  |  |  |  |  |  |  |  |  |  |
| **Wray 2018** |  |  |  |  |  |  |  |  |  |  |  |  |
