# Supplementary figures and images for: Comparing the effects of HIV self-testing to standard HIV testing for key populations: a systematic review and meta-analysis
Source: BMC Med. 2020 Dec 3;18:381. doi: 10.1186/s12916-020-01835-z (PMC7713313; doi:10.1186/s12916-020-01835-z)

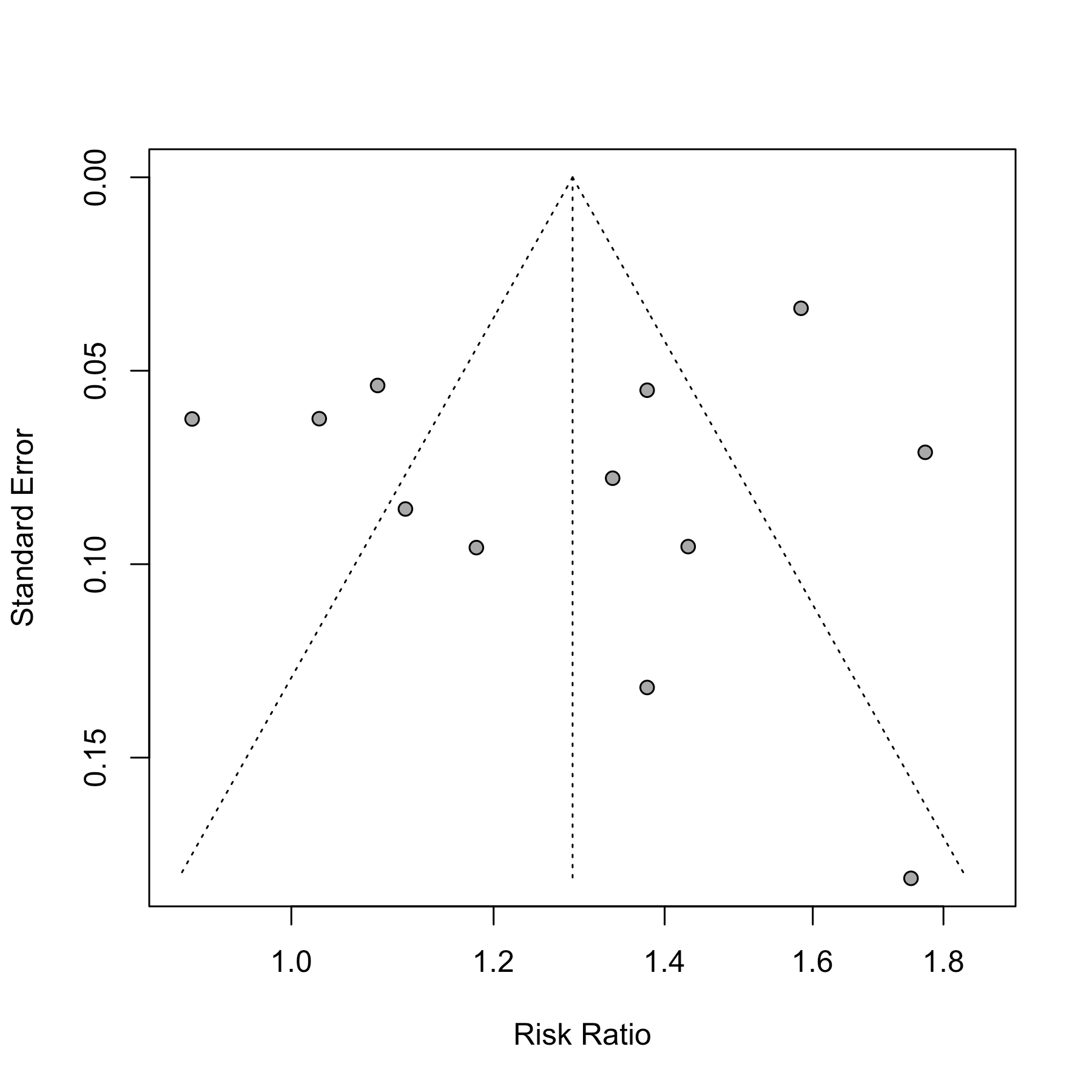

Supplement: Supplementary file 4 — Additional file 4. Uptake funnel plot. [file 12916_2020_1835_MOESM4_ESM.png]
