# Supplementary material for: Comparing the effects of HIV self-testing to standard HIV testing for key populations: a systematic review and meta-analysis
Source: BMC Med. 2020 Dec 3;18:381. doi: 10.1186/s12916-020-01835-z (PMC7713313; doi:10.1186/s12916-020-01835-z)
Supplement: Supplementary file 5 — Additional file 5. Additional forest plots. [file 12916_2020_1835_MOESM5_ESM.docx]

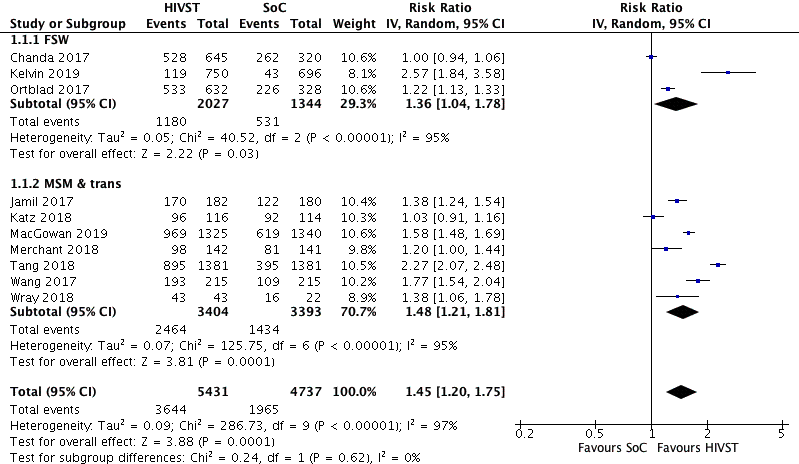


**Additional figure 1. Forest plot of HIVST uptake by sub-population**


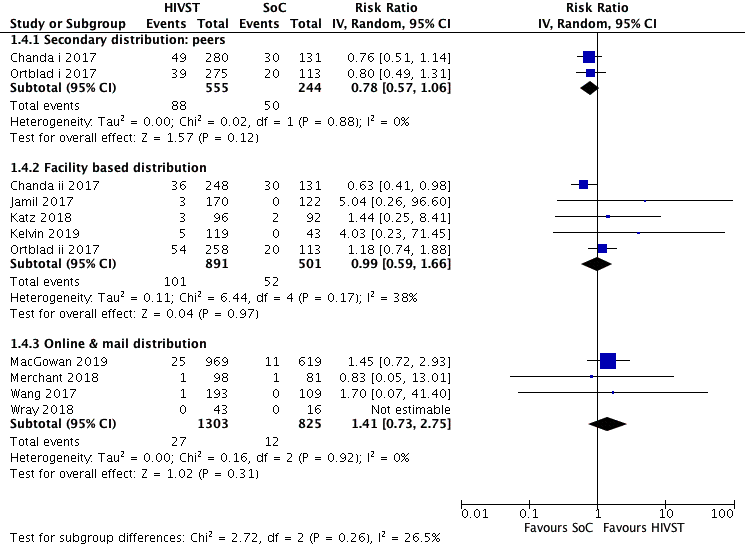


**Additional figure 2. Forest plot of positivity amongst those tested by delivery model**


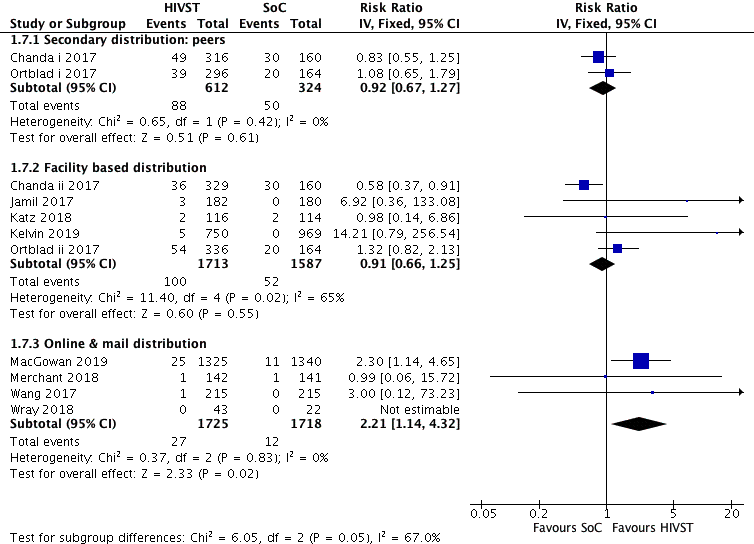


**Additional figure 3. Forest plot of positivity amongst randomised by delivery model**
